# Silica nanoparticles as a tool for fluorescence collection efficiency enhancement

**DOI:** 10.1186/1556-276X-8-146

**Published:** 2013-03-28

**Authors:** Bartosz Krajnik, Magdalena Gajda-Rączka, Dawid Piątkowski, Piotr Nyga, Bartłomiej Jankiewicz, Eckhard Hofmann, Sebastian Mackowski

**Affiliations:** 1 Institute of Physics, Nicolaus Copernicus University, Grudziadzka 5, Torun 87-100, Poland; 2 Institute of Optoelectronics, Military University of Technology, Warsaw 00-908, Poland; 3 Department of Biology and Biotechnology, Ruhr-University Bochum, Bochum 44801, Germany

**Keywords:** Silica nanoparticles, Fluorescence collection efficiency, Fluorophores, Light-harvesting complex, 87.64.K-, 87.85.jf, 77.22.-d

## Abstract

In this work we demonstrate enhancement of the fluorescence collection efficiency for chlorophyll-containing photosynthetic complexes deposited on SiO_2_ spherical nanoparticles. Microscopic images of fluorescence emission reveal ring-like emission patterns associated with chlorophyll-containing complexes coupled to electromagnetic modes within the silica nanoparticles. The interaction leaves no effect upon the emission spectra of the complexes, and the transient behavior of the fluorescence also remains unchanged, which indicates no influence of the silica nanoparticles on the radiative properties of the fluorophores. We interpret this enhancement as a result of efficient scattering of electromagnetic field by the dielectric nanoparticles that increases collection efficiency of fluorescence emission.

## Background

One of the most commonly used approaches to tune the fluorescence properties of fluorophores is to couple them to plasmonic excitations in metallic nanoparticles [1]. Large variations of shapes and sizes of metallic nanostructures provide almost infinite space for spectral engineering of optical properties of emitters, ranging from control of the fluorescence intensity, fluorescence decay dynamics, as well as the emission spectrum itself. Remarkable effects of plasmon coupling have been demonstrated on a single-molecule level, where a fluorophore was approached in a controllable way by a spherical metallic nanoparticle [2]. For large distances, the emission remained unaffected; however, as the separation decreased, a strong enhancement of the fluorescence emission has been measured. Upon further reduction of the separation between the fluorophore and metallic nanoparticle, the intensity of the fluorescence emission decreased rapidly. This result demonstrates allimportant effects of plasmon coupling in such experimental configuration, and they are associated with modifications of fluorescence quantum yield of the fluorophore, enhancement of its excitation rate, and quenching due to nonradiative energy transfer to the metallic nanoparticle. As these processes compete against each other, in order to achieve strong enhancement of the fluorescence intensity, it is crucial to put attention to the geometry of the hybrid plasmonic nanostructure, in particular to the control of the separation between fluorophores and metallic nanoparticles.

The concept of coupling fluorescent molecules with metallic nanoparticles has been then extended to other nanostructures, such as multichromophoric photosynthetic pigment-protein complexes [3]. In this case the distance between metallic nanoparticles and proteins was controlled via silica layers with defined thickness. It has been shown that depending upon actual arrangement of the hybrid nanostructure, it is possible to obtain strong enhancement of the absorption rate [4] or increase of the fluorescence rate [5] in such a system. Importantly, in order to determine which of the two processes is responsible for the observed enhancement of the fluorescence, it is necessary to combine standard steady-state experiment with time-resolved fluorescence spectroscopy [6].

Another method applied to increase the fluorescence of molecules is based on applying dielectric nanospheres [7]. Such structures feature strong magnetic resonances, thus can be used for changing emission of molecules that feature not only magnetic but also electric dipole moment [8]. On the other hand, such nanoparticles are characterized with high refractive index; therefore, placing them between collection optics and emitters results in improvement of optical resolution and collection efficiency [9-14]. One of the examples is a solid immersion lens [12], frequently a hemispherical macroscopic lens made of high-refractive-index glass (*n* = 1.84 and *n* = 1.69 in [12]), using of which can yield a significant (factor of *n*) increase of the optical resolution. It has also been shown that solid immersion lenses can be applied for high-resolution imaging of semiconductor structures at cryogenic temperatures [14]. On the other hand, application of dielectric nanoparticles has been discussed in the context of enhancing optical response in the infrared as well as in the visible spectral range. It has been shown that for the emission of a single molecule placed onto a surface of a dielectric microsphere, it is possible to observe up to fivefold enhancement of the fluorescence intensity when such a structure is illuminated with a Gaussian beam [9]. This effect was attributed to strong confinement of the electromagnetic field near the particle. Importantly, dielectric nanostructures have been also suggested as an efficient source of absorption enhancement in solar cell architectures due to creation of whispering gallery modes by properly chosen illumination [10]. All these findings point towards a broad range of possibilities of introducing spherical dielectric nanoparticles for controlling the optical properties in many applications. In addition, it has been shown that such nanoparticles can be coated with metallic islands for enhanced Raman scattering [15,16].

In this work we focus on hybrid nanostructures composed of photosynthetic complexes and spherical silica nano(micro)spheres. By fluorescence microscopy and spectroscopy, we demonstrate almost a threefold enhancement of the fluorescence emission of the photosynthetic complexes located near the dielectric spheres. The results suggest that the enhancement factor depends upon the size of nanoparticles. The spectral shape as well as dynamic behavior of the emission remains unchanged upon coupling with the nanospheres; therefore, we attribute the observed enhancement as being due to enhanced efficiency of light collection from molecules in the vicinity of the silica nanoparticles.

## Methods

Peridinin-chlorophyll-protein (PCP) photosynthetic molecules were obtained according to the protocol by Miller et al. [17]. Briefly, PCP apoprotein in 50 mM Tris-HCl (pH 8.0) solution was added to 25 mM tricine and 10 mM KCl (pH 7.6), mixed with a stoichiometric amount of PCP pigments dissolved in ethanol. The sample was held in 4°C for 72 h. Reconstituted samples were equilibrated to 5 mM tricine with 2 mM KCl (pH 7.6) by passage through a PD-10 column and bound to a column of DEAE Trisacryl (Sigma-Aldrich, St. Louis, MO, USA). Reconstituted PCP was then removed with 5 mM tricine with 2 mM KCl (pH 7.6) containing 0.06 M NaCl. The protein solution was characterized by absorption and fluorescence spectroscopy.

All reagents for silica nanoparticle synthesis were purchased and used as received from the indicated suppliers: nitric acid, hydrochloric acid, ammonium hydroxide (25%), and glucose from Chempur (Karlsruhe, Germany); potassium hydroxide and ethanol from POCh (Gliwice, Poland); tetraethylorthosilicate from Sigma-Aldrich (St. Louis, MO, USA); and silver nitrate from Lach-ner (Neratovice, Czech Republic). Deionized water was purified to a resistance of 18.2 MΩ (HLP 5UV System, Hydrolab, Hach Company, Loveland, CO, USA) and filtered using a 0.2-μm membrane filter to remove any impurities. All glassware and equipment were first cleaned in an aqua regia solution (3:1, HCl/HNO_3_) and rinsed with ultrapure water prior to use. All solutions were prepared under stirring and/or sonication, using 18.2 MΩ cm of ultrapure water. Silica particles with diameters of 250 nm to 1.1 μm and low dispersities were prepared using a variation of the method developed by Stöber et al. [18]. The obtained nanoparticles were characterized by scanning electron microscopy and absorption spectroscopy.

The samples for fluorescence measurements were prepared by spin-coating the solution of silica nanoparticles onto a clean microscope cover slip. For that purpose, equal volumes of nanoparticle solution were mixed with PCP solution at a concentration of 2 μg/mL. After that, a solution of the PCP complexes was deposited on the nanoparticles. Alternative approach of mixing both samples prior to spin-coating was used, and the results were qualitatively identical.

Absorption spectra were recorded on a Varian-Cary 50 UV-visible spectrophotometer (Palo Alto, CA, USA). Steady-state fluorescence measurements were performed using a FluoroLog 3 spectrofluorometer (Jobin Yvon) equipped with a double grating monochromator. A xenon lamp source was used for excitation, and the signal was detected with a thermoelectrically cooled photomultiplier tube with a dark current less than 100 cps.

Fluorescence intensity maps were measured with a Nikon Eclipse Ti inverted wide-field microscope (Tokyo, Japan) equipped with Andor iXon Du-888 EMCCD (Belfast, UK) with a dark current 0.001 e-/pix/s at −75°C. The excitation was provided by a LED illuminator with a central wavelength of 480 nm. In order to narrow down the excitation beam spectrally, we used in addition a band-pass filter, FB480-10. The beam was reflected with a dichroic beam splitter (Chroma 505DCXR, Rockingham, VT, USA) to the microscope objective (Plan Apo, ×100, oil immersion, Nikon). The excitation power of illumination was about 60 μW. Fluorescence intensity maps of the PCP complexes were obtained by filtering the spectral response of the sample with a band-pass filter (Chroma HQ675-20).

Measurements of fluorescence spectra and decays were carried out using our home-built fluorescence microscope based on the Olympus long working distance microscope objective LMPlan ×50, NA 0.5 [19]. First of all, silica nanoparticles were localized on the sample surface using the scanning mode of the microscope, and then from selected points corresponding to the emission of the PCP complexes placed close to the silica nanoparticles, spectra and decays were measured. For the reference, we also measured a similar set of data from areas away from the nanoparticles. The excitation was provided by a picosecond pulsed laser at 485 nm with an excitation power of 60 μW at a repetition rate of 50 MHz. The fluorescence spectra were measured by dispersing the emission using an Amici prism and detecting the spectrum with a CCD detector (Andor iDus DV 420A-BV). Fluorescence decays were obtained using a time-correlated single-photon counting approach, with a fast avalanche photodiode as the detector. The emission of the PCP complexes was extracted using a band-pass filter, HQ675-20.

## Results and discussion

**Figure 1:**
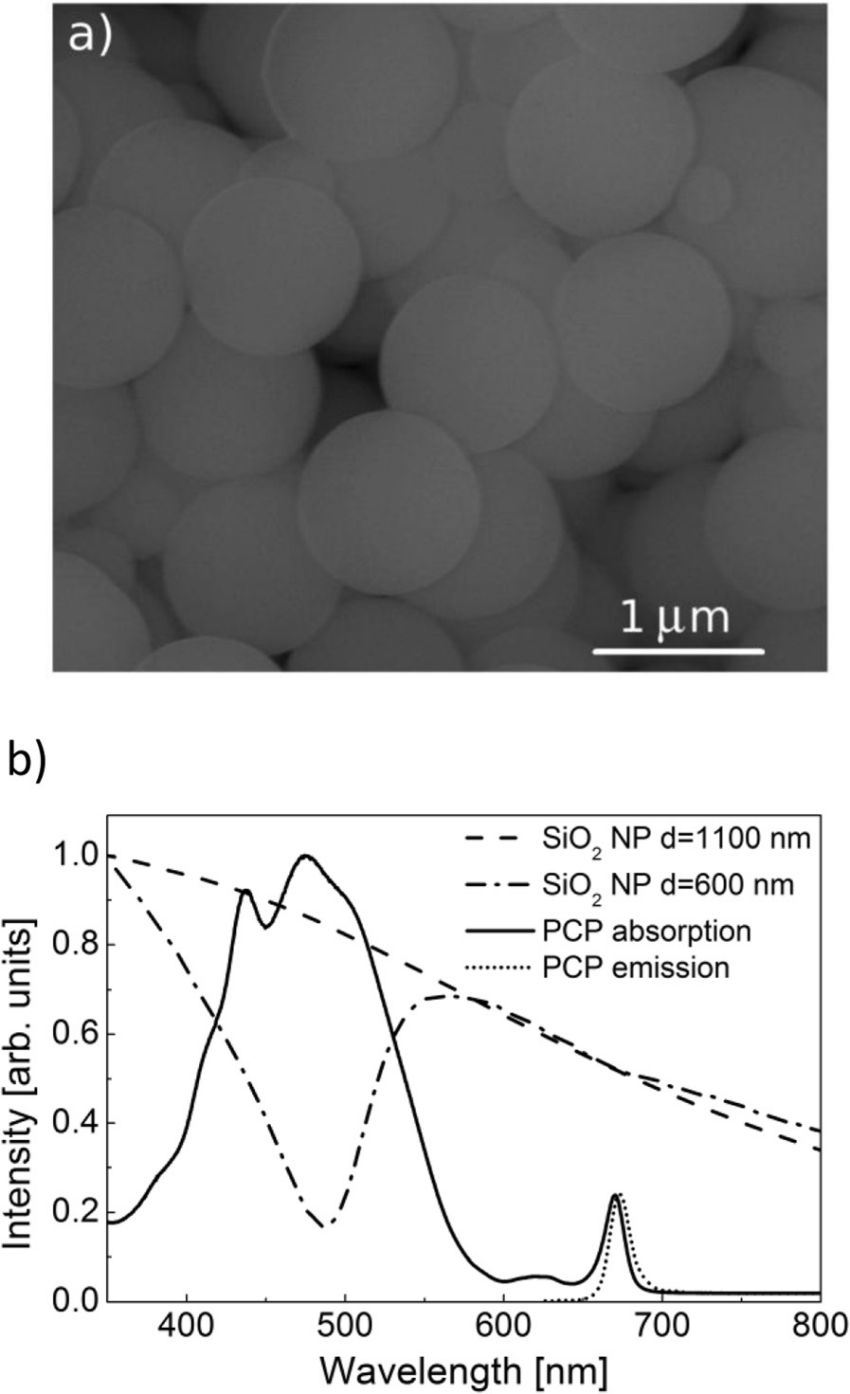
**Scanning electron microscopy image and optical spectra of the silica nanoparticles.** (**a**) Scanning electron microscopy image of the silica nanoparticles with a diameter of 1,100 nm. (**b**) Optical spectra of silica nanoparticles with diameters of 600 nm (dash-dot) and 1,100 nm (dash) compared to absorption spectrum of the PCP complex solution (solid) as well as its fluorescence (dot).

Figure 1 shows the scanning electron microscopy image of the silica nanoparticles with a nominal diameter of 1,100 nm. The sample is highly homogeneous, although some of the nanoparticles feature smaller sizes. The structural data are accompanied with the extinction spectrum of the 1,100- (dashed line) and 600-nm (dash-dot line) particles shown in Figure 1b). The data were normalized in order to facilitate better comparison. The spectrum obtained for the larger particles decreases smoothly and monotonously towards longer wavelengths, while the spectrum obtained for the 600-nm particles features a dip in intensity around 500 nm and a long tail towards longer wavelength region. The absorption spectrum of the PCP complexes is displayed for comparison in Figure 1b (solid line). The major absorption band spans from 400 to 550 nm and is attributed predominantly to absorption of peridinins in the complex [20]. The absorption of the chlorophyll molecules appears around 440 and 660 nm. The emission spectrum of the PCP complexes shown by a dotted line in Figure 1b features a single band at 670 nm [5], which is due to recombination in chlorophyll molecules. We note that the emission of the PCP complexes overlaps with the extinction spectra of the silica nanoparticles.

**Figure 2:**
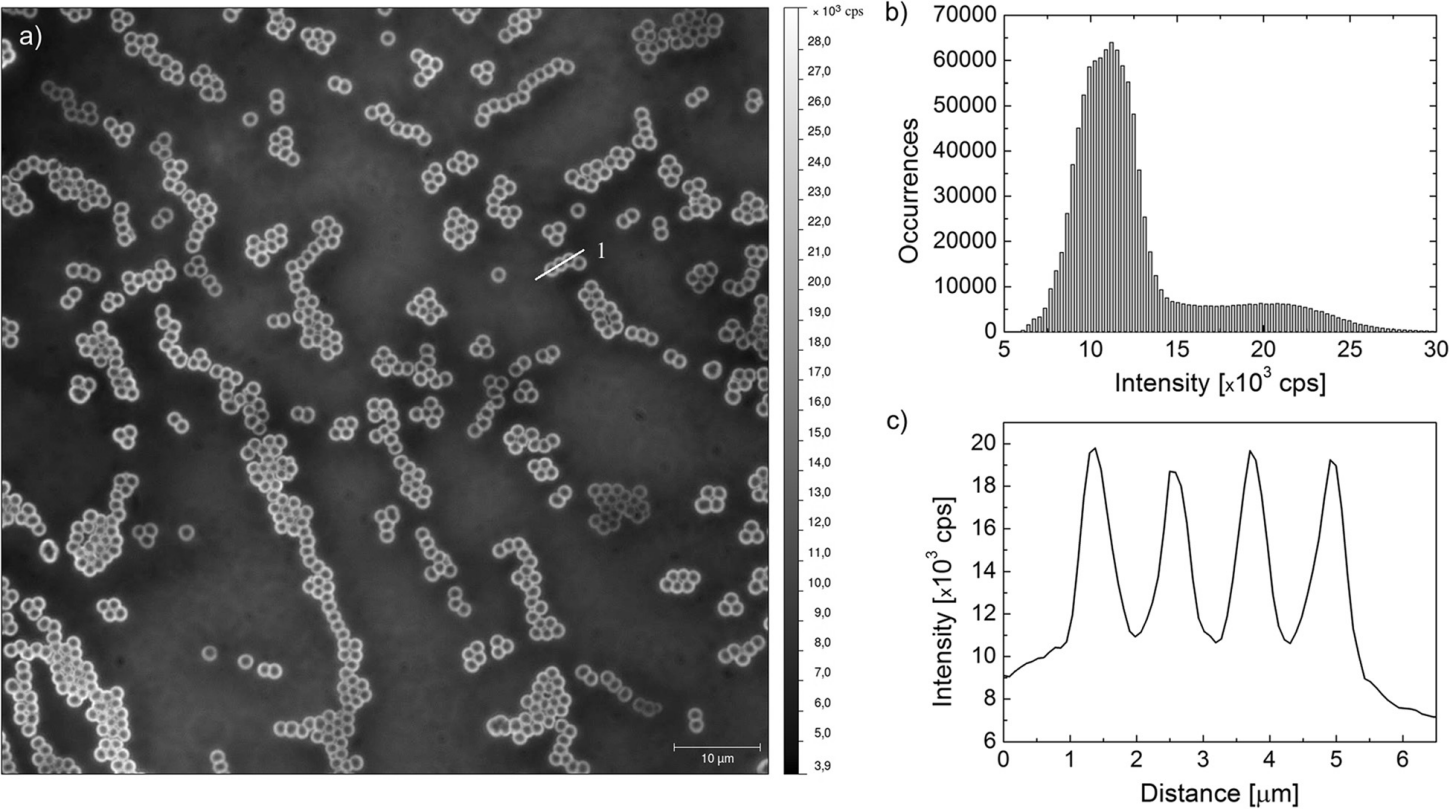
**Wide-field fluorescence image of the PCP complexes on 1.1-μm-diameter silica nanoparticles and their fluorescence intensity.** (**a**) Wide-field fluorescence image of the PCP complexes deposited on silica nanoparticles with a diameter of 1.1 μm. Excitation wavelength was 480 nm. (**b**) Histogram of the fluorescence intensity calculated from the wide-field fluorescence image. (**c**) Cross section of the fluorescence intensity obtained for the three nanoparticles shown in Figure 2a.

The method used for sample preparation results with the PCP complexes being either very close to the nanoparticles or completely away. In this way, we can determine the fluorescence intensity for both sets of PCP complexes in the same sample. Typical fluorescence image of the PCP complexes coupled to the silica nanoparticles with a diameter of 1.1 μm is shown in Figure 2. The 90 × 90 μm image obtained by wide-field microscopy technique features many almost identical ring-shaped structures, with only a few exceptions. Such a high uniformity indicates - in accord with the structural data - high homogeneity of the silica nanoparticles used for preparing the hybrid nanostructure. Many of the nanoparticles are connected together; however, uniform intensities suggest that the nanoparticles form a sub-monolayer on the cover slip surface. The observed rings are due to the PCP complexes that are close to the silica nanoparticles. The emissions from such complexes exhibit considerably higher intensity as compared to those from the PCP complexes that are far away from the nanoparticles. The difference is visualized in Figure 2b, where we plot a histogram of intensities obtained for a fluorescence image similar to the one shown in Figure 2a. The distribution is of a quasi-bimodal character. The subset around 10^4^ counts per second corresponds predominantly to the PCP complexes that are away from the silica nanoparticles; on the other hand, the distribution around 2.2 × 10^4^ counts per second is attributable to the PCP complexes that are in the vicinity of the silica nanoparticles and whose fluorescence is more efficiently collected by the resulting optical system. It is also instructive to determine the intensity profile for the PCP complexes coupled to silica nanoparticles that are in touch with each other, similarly to what is shown in Figure 2a (drawn by a white line). In this case we find three nanoparticles in line, and all of them feature enhancement of the emission of the PCP complexes. The intensity cross section of the fluorescence intensity obtained for these three nanoparticles is shown in Figure 2c. It is evident that the intensity of the emission of the PCP complexes at the edges of the silica nanoparticles is constant, regardless of whether the PCP complexes are at the edge of a nanoparticle or in between two nanoparticles. In this case a twofold enhancement of the fluorescence intensity is observed. Such a behavior is qualitatively different from the one demonstrated frequently for closely placed metallic nanoparticles, where a so-called hot spot can be formed, where the total fluorescence intensity can be considerably higher than for a single nanoparticle. The difference confirms that the mechanism responsible for the fluorescence enhancement observed for a hybrid nanostructure assembled from dielectric spheres and photosynthetic complexes has another origin.

**Figure 3:**
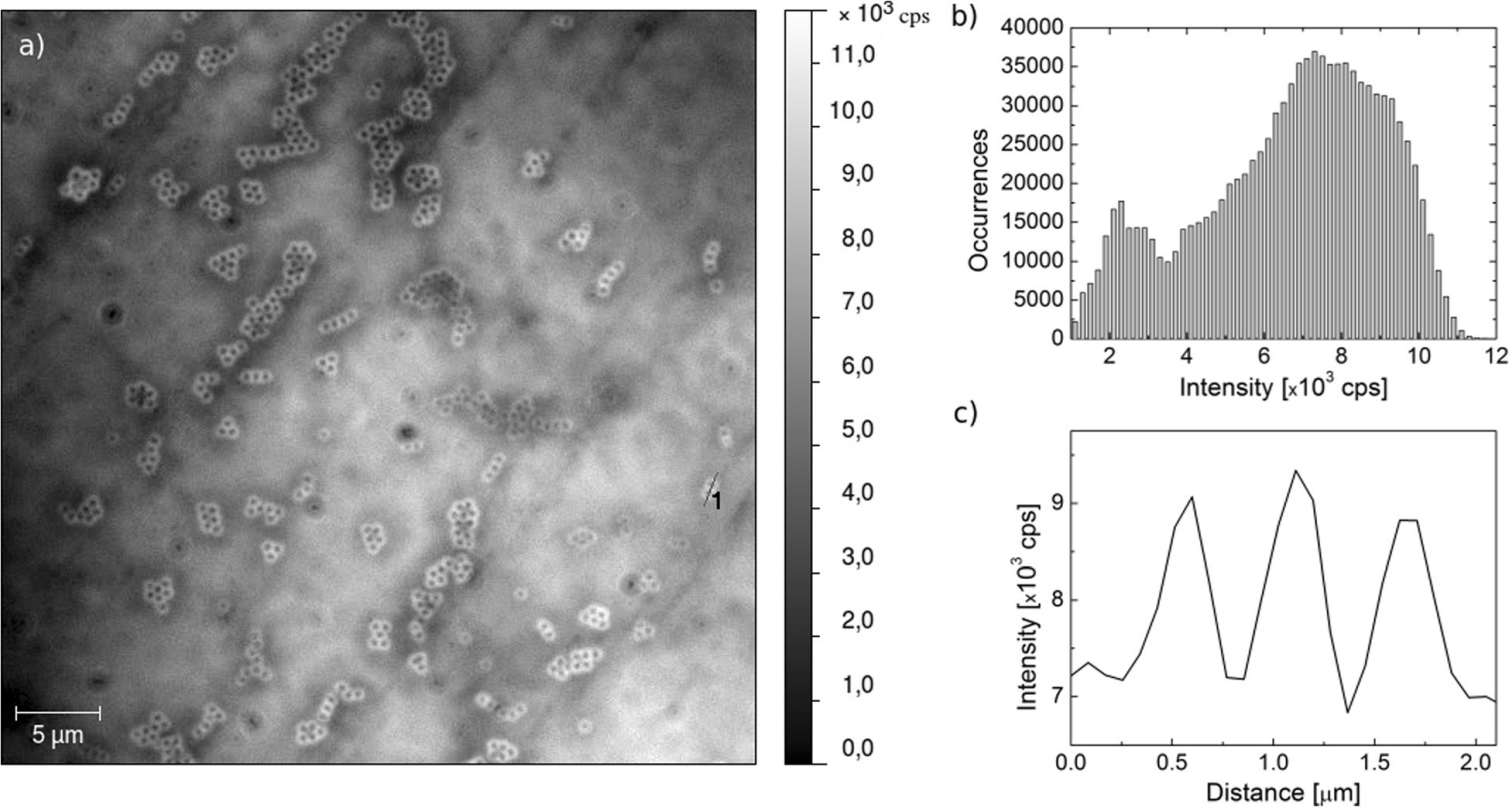
**Wide-field fluorescence image of the PCP complexes on 0.6-μm-diameter silica nanoparticles and their fluorescence intensity.** (**a**) Wide-field fluorescence image of the PCP complexes deposited on silica nanoparticles with a diameter of 0.6 μm. Excitation wavelength was 480 nm. (**b**) Histogram of the fluorescence intensity calculated from the wide-field fluorescence image. (**c**) Cross section of the fluorescence intensity obtained along the black line 1 for the three nanoparticles shown in Figure 3a.

The enhancement factor of the fluorescence depends upon the size of dielectric particles. In Figure 3, we show a dataset similar to the one discussed above, but obtained for smaller particles, having a diameter of 600 nm. In the fluorescence map (Figure 3a), we also can see ring-like emission patterns that originate from the PCP complexes placed in the vicinity of the silica spheres. Analogous analysis has been carried out for this structure in order to estimate the influence of silica nanoparticles upon the collection efficiency of the fluorescence. In this case the fluorescence map shows however substantial inhomogenities of the emission intensity of the PCP complexes away from the nanoparticles, as evidenced by the intensity histogram (Figure 3b). An intensity cross section displayed in Figure 3c features the increase of the intensity at the edges of the nanoparticles; however, the scale of the enhancement is lower than that in the case of 1,100-nm particles. Although the particle doublet shown in Figure 3c might be on the lower side of enhancement factors measured for this structure, we have not observed cases with the increase larger than twofold. The comparison between the fluorescence images obtained for the PCP complexes deposited on 1,100- and 600-nm silica spheres suggests that the enhancement of collection efficiency could depend upon the diameter of dielectric particles, but a clear answer can be given perhaps after performing single-molecule fluorescence studies in this geometry.

**Figure 4:**
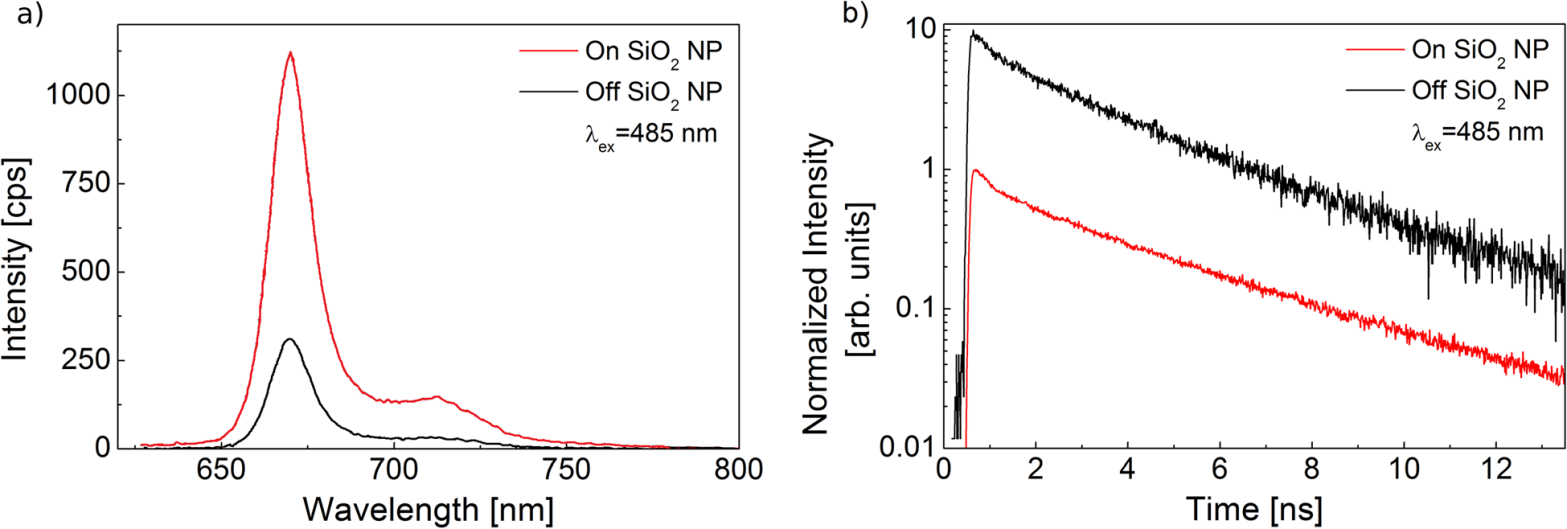
**Emission spectra and fluorescence decay curves of the PCP complexes.** (**a**) Emission spectra of the PCP complexes deposited on (red) and off (black) silica nanoparticles. (**b**) Fluorescence decay curves of PCP deposited on (red) and off (black) silica nanoparticles. The excitation wavelength for both experiments was 480 nm. The transients are normalized, and the one measured for the PCP complexes off the silica nanoparticles was shifted vertically (multiplied by 10) for clarity.

In order to verify that the emission observed using a wide-field microscope is indeed associated with the PCP complexes, we obtain fluorescence spectra and decay curves for an identically prepared structure. The confocal image, in contrast to the wide-field image, consists of bright spots spread over otherwise quite uniform background. We attribute the spots to the emission of the PCP complexes close to the silica nanoparticles, and the background originates from the PCP complexes placed far away from the nanoparticles. The absence of the ring-like structure on the confocal images is a result of much lower numerical aperture of the collection optics (0.5 vs. 1.4), which results in much lower spatial resolution of the experiment. After collecting such a confocal image, we measured spectra and decays for several tens of bright spots and compare the result with the data obtained for the areas free of the nanoparticles. An example of the results is displayed in Figure 4. The comparison of the fluorescence spectra measured for the PCP complexes on and off the nanoparticles (Figure 4a) indicates that the coupling with the nanoparticles leaves no effect upon the spectral shape of the emission. The only impact concerns the total fluorescence intensity and the result that is intact with the observations made by wide-field microscopy. The average enhancement of the fluorescence emission obtained from this comparison is equal to 3. Similarly, the transient behavior of the fluorescence intensity is also identical for the PCP complexes placed on and off the silica nanoparticles (Figure 4b). Unchanged lifetimes indicate that the interaction between the nanoparticles and the photosynthetic complexes induces no changes in the radiative properties of the chlorophyll molecules that are responsible for the fluorescence emission.

## Conclusions

We find that coupling of photosynthetic, chlorophyll-containing complexes with dielectric silica nanoparticles leads to an enhancement of the fluorescence emission. The interaction leaves no measurable effect on the shape of the emission as well as on the transient behavior of the fluorescence. We conclude that the effect of fluorescence enhancement originates from high scattering of electromagnetic field by dielectric nanoparticles that leads to improvement of the collection efficiency. Although several aspects of the results described in this work are still to be understood completely, the experiment is a vital step towards assembling functional nanostructures that exploit enhancement effects associated with dielectric nanoparticles.

## Abbreviations

EMCCD: electron-multiplying charge-coupled device; PCP: peridinin-chlorophyll-protein.

## Competing interests

The authors declare that they have no competing interests.

## Authors’ contributions

BK and DP carried out the fluorescence experiments and analyzed the results. MG-R, PN, and BJ synthesized the dielectric nanoparticles used in this work. EH provided the reconstituted photosynthetic complexes. PN, BJ, and SM designed the study and coordinated the research and collaboration between the groups. BJ and SM wrote the manuscript. All authors read and approved the final manuscript.
